# Interactions of human microglia cells with Japanese encephalitis virus

**DOI:** 10.1186/s12985-016-0675-3

**Published:** 2017-01-14

**Authors:** Nils Lannes, Viviane Neuhaus, Brigitte Scolari, Solange Kharoubi-Hess, Michael Walch, Artur Summerfield, Luis Filgueira

**Affiliations:** 1 Department of Medicine, Unit of Anatomy, University of Fribourg, Route Albert-Gockel 1, Fribourg, Switzerland; 2 Institute of Virology and Immunology, Sensemattstrasse 293, Mittelhäusern, Switzerland; 3 Department of Infectious Diseases and Pathobiology, Vetsuisse Faculty, University of Bern, Langassstrasse 122, Bern, Switzerland

**Keywords:** Human microglia, Japanese encephalitis virus, Inflammation, Chemokine ligand-receptor, Viral transmission

## Abstract

**Background:**

Japanese encephalitis virus (JEV) is a neurotropic flavivirus causing mortality and morbidity in humans. Severe Japanese encephalitis cases display strong inflammatory responses in the central nervous system and an accumulation of viral particles in specific brain regions. Microglia cells are the unique brain-resident immune cell population with potent migratory functions and have been proposed to act as a viral reservoir for JEV. Animal models suggest that the targeting of microglia by JEV is partially responsible for inflammatory reactions in the brain. Nevertheless, the interactions between human microglia and JEV are poorly documented.

**Methods:**

Using human primary microglia and a new model of human blood monocyte-derived microglia, the present study explores the interaction between human microglia and JEV as well as the role of these cells in viral transmission to susceptible cells. To achieve this work, vaccine-containing inactivated JEV and two live JEV strains were applied on human microglia.

**Results:**

Live JEV was non-cytopathogenic to human microglia but increased levels of CCL2, CXCL9 and CXCL10 in such cultures. Furthermore, human microglia up-regulated the expression of the fraktalkine receptor CX_3_CR1 upon exposure to both JEV vaccine and live JEV. Although JEV vaccine enhanced MHC class II on all microglia, live JEV enhanced MHC class II mainly on CX_3_CR1^+^ microglia cells. Importantly, human microglia supported JEV replication, but infectivity was only transmitted to neighbouring cells in a contact-dependent manner.

**Conclusion:**

Our findings suggest that human microglia may be a source of neuronal infection and sustain JEV brain pathogenesis.

**Electronic supplementary material:**

The online version of this article (doi:10.1186/s12985-016-0675-3) contains supplementary material, which is available to authorized users.

## Background

Japanese encephalitis (JE) is an acute inflammatory disease of the central nervous system (CNS) caused by the neurotropic flavivirus JE virus (JEV). JEV is a single stranded positive sense RNA virus endemic in the Asia-Pacific region, including China, India and northern Australia [1]. JEV is transmitted by mosquito vectors via a zoonotic cycle involving pigs as amplifying and water birds as reservoir host, the latter do not typically develop illness upon JEV infection [2]. Humans are considered to be dead-end hosts, since low viremia does not allow further virus transmission [1].

Markedly, competent vectors for JEV have been recently identified in Germany [3] and the ability of JEV to persist and transmit between pigs in absence of mosquitos [4] are increasing risks of virus spread and persistence in regions with more moderate climate and becoming a worldwide public health concern. While less than 1% of JEV infected patients develop JE, it is estimated ~70,000 annual human symptomatic JE cases to happen, where 25-30% are fatal and 50% of surviving patients develop permanent neurological damage [5, 6]. In regions at risk, vaccination programs are available [5].

By an unknown mechanism, JEV invades the CNS infecting and killing neurons with a specific tropism for developing neurons [7, 8]. In brain autopsies of fatal JE patients, JEV–infected neurons are localized in the thalamus, in the brainstem, as well as in the hippocampus, which are areas of neuronal turn-over, even in adults [9]. Accumulating evidences highlight the prominent role of the cells of the macrophage lineage in JEV pathogenesis. In fact, human blood monocytes survive from productive infection by JEV and can maintain infectious virus for 5 days [10]. Human blood monocyte-derived dendritic cells (MoDC) and monocyte-derived macrophages (MDM) support virus replication in vitro [11, 12].

Microglial cells are the unique resident immune cells of the nervous system, which populate the brain during early development, but can also derive from blood monocytes after birth under specific conditions [13]. Microglia are involved in immune surveillance of the CNS and have potent migratory functions driven by chemokine ligands/receptors interactions, as well as phagocytic and antigen presentation abilities [14, 15]. Mouse microglia are activated and produce numbers of pro-inflammatory factors upon JEV infection in vivo and in vitro [16]. Moreover, mouse microglia are productively infected by JEV in vitro and are proposed to play a role in long-lasting infection [17].

Despite differences between humans and most of animals, rodent models are widely used in the laboratory to characterize and understand JEV pathogenicity. The present study explores the interactions between human microglia and JEV. To achieve this work, vaccine-containing inactivated JEV and two live JEV strains were applied in-vitro on human primary microglia and human monocyte-derived microglia. Our results show that human microglia were activated upon JEV exposure, without affecting viability. Indeed, JEV-exposed human microglia acquired an inflammatory state, characterized by increased levels of chemokine ligands such as CCL2, CXCL9 and CXCL10 and increased expression of the fraktalkine receptor CX_3_CR1. Furthermore, JEV enhanced MHC class II expression. Finally, human microglia supported viral replication but cell-cell contact was required for viral transmission to other cells. The present study demonstrates the implication of human microglia in inflammatory responses and virus propagation upon exposure to JEV.

## Methods

### Authorization

Authorization (number A130522) was obtained from the Federal Office for the Environment (FOEN, Bern, Switzerland) for collection of human samples and manipulation of the various cells and viruses. All samples were analysed anonymously.

### Preparation of human microglial cells and cell culture

Human primary microglia cells (BdMG) were isolated from the cortex of brains derived from an anonymous donor of the body donation program of the University of Fribourg according to a newly established protocol from [18]. Briefly, the brain was excised within 8 h post-mortem. The 20 mL of minced cortex tissue was digested at room temperature for 24 h under gentle shaking in 75 cm^2^ culture flasks (Corning Incorporated, Corning, NY), containing 30 mL Roswell Park Memorial Institute-1640 (RPMI-1640) GlutaMAX™-I medium supplemented with 2x antibiotics/antimycotic and 0.5x trypsin/EDTA (all from Life Technologies). The resulting single cells in suspension were further enriched through filtering through Smarttrainer (100 μm, Miltenyi Biotec GmbH, Bergisch-Gladbach, Germany) and Ficoll-Paque density gradient centrifugation (1.077 g/L, Amersham Pharmacia Biotech AG, Dubendorf, Switzerland). The cells were then cultured in RPMI-1640 GlutaMAX™-I medium supplemented with 1x antibiotic/antimycotic and 5% v/v human serum (HS) (heat inactivated, obtained from Don du sang, Lausanne, Switzerland) at 37 °C and 5% CO_2_.

Human blood monocyte-derived microglia (M-MG) were generated from buffy coats of anonymous healthy donors, alternatively obtained from Don du sang (Lausanne, Switzerland) and Blutspendedienst (Bern, Switzerland), as previously described [19]. Briefly, human peripheral blood mononuclear cells (PBMC) were isolated from buffy coat after Ficoll-Paque density gradient centrifugation. PBMC were cultured in 25 cm^2^ culture flasks (Corning, Incorporated) in RPMI-1640 GlutaMAX™-I medium supplemented with 1x antibiotic/antimycotic for at least 2 h allowing adherence of cells. After adherence, non-adherent cells and contaminants were washed away. For differentiation toward microglia, adherent cells, mainly monocytes, were cultured in complete medium consisting of RPMI-1640 GlutaMAX™-I medium supplemented with 1x antibiotic/antimycotic and the following bioactive human recombinant cytokines (purchased from Miltenyi Biotec GmbH, Bergisch-Gladbach, Germany) at the indicated concentration: granulocyte macrophage colony-stimulating factor (GM-CSF) (10 ng/mL), macrophage colony-stimulating factor (M-CSF) (10 ng/mL), nerve growth factor (NGF)-β (10 ng/mL) and CC chemokine ligand 2 (CCL2) (50 ng/mL) at 37 °C and 5% CO_2_ for 7–10 days.

Baby Hamster Kidney-21 cells (BHK-21) ([C-13], ATCC, Wesel, Germany) were cultured in Glasgow’s Minimum Essential Medium (GMEM) (Life Technologies, Zug, Switzerland) supplemented with 5% v/v Fetal Bovine Serum (FBS) (Biowest, Nuaillé, France) and Tryptose Phosphate Broth solution (Sigma-Aldrich, Saint Louis, MO) at 37 °C and 5% CO_2_.

### Viruses: source, propagation and titration

JEV vaccine, manufactured by Intercell AG (Vienna, Austria) and commercialized under the name Ixiaro^®^ by Novartis (Basel, Switzerland), was obtained from the Inselspital Pharmacy (Bern, Switzerland). One dose of Ixiaro^®^ consists of 6 μg of formalin-inactivated SA_14_-14-2 isolate protein (Vero cell-derived attenuated JEV isolate) and aluminium hydroxide adjuvant (0.25 mg Al^3+^) in 0.5 mL suspension PBS solution [20]. Filtered suspension of aluminium hydroxide (0.5 mg/mL) (Sigma-Aldrich) in PBS was used as control.

Nakayama and TC362 isolates (National collection of pathogenic viruses, NCPV, Salibury, UK) were propagated and titrated in BHK-21. Briefly, 80% confluent BHK-21 monolayer cell culture was infected with JEV suspended in RPMI-1640 GlutaMAX™-I medium supplemented with 2% FBS. Cells were cultured until cytopathogenic effects appear (approx. 36–48 h). Remaining cells were disrupted by freezing and cell soup was centrifuged at 3000 g at 4 °C for 30 min to eliminate cell debris. Viral titres were determined by end-point titration on BHK-21 cells. To this end, 10-fold serial dilutions of the virus stocks were applied on cell cultures for 36–48 h at 37 °C, 5% CO_2_. Then, intracellular viral particles were detected with the pan-immune anti-flavivirus antibody (mouse clone ATCC-HB-112 D1-4G2-4-15 hybridoma, ATCC, Wesel, Germany) followed by peroxidase enzymatic reaction. As control, mock antigen was prepared from uninfected BHK-21 cells in the same manner as JEV.

### Treatment of human microglial cells with JEV and co-culture with BHK-21 cells

In experiments, human microglia were cultured in serum-free RPMI-1640 GlutaMAX™-I medium. Addition of serum abrogated the responsiveness of human microglia (data not shown). At a concentration of 5x10^5^ cells/mL, human microglia were treated either with JEV vaccine or live JEV in RPMI-1640 GlutaMAX™-I medium at 37 °C and 5% CO_2_. After treatment, cells and supernatants were separately collected for further analysis. In some experiments, supernatants were stored at -80 °C until further experiments.

In some experiments, JEV-treated human microglia: BHK-21 cells (10: 1) were co-cultured in serum-free RPMI-1640 GlutaMAX™-I medium for 2 days at 37 °C in 5% CO_2_. Before addition of BHK-21 cells, JEV-treated human microglia were intensively washed with fresh medium and last washing was verified negative for infectious JEV. On one hand, human microglia and BHK-21 cells were cultured in cell-cell contact condition by addition of BHK-21 cells on top of human microglia. On another hand, human microglia and BHK-21 cells were separated using transwell insert (0.4 μm polyester membrane, Corning, Incorporated), allowing the passage of small material such as viral particles but not cells. In these conditions, the lower chamber contained human microglia and the upper chamber contained BHK-21 cells.

### Bright field microscopy

Cells in culture were observed with an Eclipse TS1000 light microscope (Nikon AG, Egg, Switzerland) and photographed using a EOS 600D Camera (Canon SA, Wallisellen, Switzerland).

### Transmission electron microscopy

Cells were successively fixed with 0.05% glutaraldehyde and 4% paraformaldehyde. Then, cells were embedded in LR White medium grade acrylic resin (SPI Supplies, West Chester, PA). Ultrathin sections (0.05 μm thick) contrasted with negative stain uranyl acetate solution (Sigma-Aldrich) and analysed using a CM100-Biotwin transmission electron microscope (Philips SA, Zürich, Switzerland).

### Antibodies and flow cytometry

For phenotyping, fluorescent-labelled anti-human antibodies against the following cell surface markers were employed and purchased from BD Biosciences (San Jose, CA) otherwise stated: CCR1-Alexa Fluor 647 (AF647) (CD191, clone 53504), CCR2-AF647 (CD192, clone 48607), CCR3-R-Phycoerythrin (R-PE) (CD193, clone 5E8), CCR4-Peridin chlorophyll protein (PerCP)-Cy5.5 (CD194, clone 1G1), CCR5- Allophycocyanin (APC)-Cy7 (CD195, clone 2D7/CCR5), CXCR1-Fluorescein isothiocyanate (FITC) (CD181, clone 5A12), CXCR2-FITC (CD182, clone 6C6), CXCR3-R-PE-Cy5 (CD183, mouse clone 1C6/CXCR3), CXCR4-R-PE-Cy5 (CD184, mouse clone 12G5), CXCR5-AF488 (CD185, clone RF8G2), CX_3_CR1-R-PE (clone 2A9-1 from Miltenyi Biotec GmbH, Bergisch-Gladbach, Germany) and MHC class II-APC (HLA-DR, clone G46-6). Corresponding fluorescent-labelled isotype antibody controls were selected according to manufacturer’s recommendations. In addition, MHC class I (mouse clone PT85A, IgG2a, VRMD Inc, Pullman, WA) and goat anti-mouse all IgG fluorescent-labelled with R-PE (BD Biosciences) were used.

Viral particles were detected intracellularly using the pan-immune anti-flavivirus antibody (ATCC) as primary antibody. Secondary antibody was the goat anti-mouse IgG1 fluorescent-labelled with AF647 (Life Technologies).

Concentrations for use of the antibodies were optimized in our laboratory. Cells were analysed using multi-colour flow cytometry (MACSQuant instrument from Miltenyi Biotech and BD FACSCanto II instrument from BD Biosciences). Data were analysed using FlowJo Software (Data analysis Software, Ashland, OR).

### Determination of cell death by apoptosis

Cells were labelled with Annexin-V-APC (Affimetrix eBioscience, Vienna, Austria) [21]. Cells were analysed using single colour flow cytometry.

### Chemokine beads assay

Levels of the human chemokines CCL2, CCL5, CXCL8, CXCL9 and CXCL10 were quantified in supernatants using a human chemokine beads assay (Human Chemokine Kit, BD Biosciences) following manufacturer’s recommendations. Beads were analysed using multi-colour flow cytometry and concentrations of chemokines were determined by comparison with the standard material included in the kit.

### Real-time RT-PCR

JEV RNA was detected by real-time RT-PCR assay. RNA from supernatant and cells were separately extracted using TRIzol method (Life technologies) supplemented with glycogen (Ambion). As internal control, in vitro enhanced green fluorescent protein (EGFP) transcript was added to samples in TRIzol before further steps of RNA extraction. EGFP primers and probe were specific for EGFP transcript sequence: forward primer 5′-GGGCACAAGCTGGAGTACAAC-3′, reverse primer 5′-CACCTTGATGCCGTTCTTCTG-3′ and probe YYE-acaacagccacaacgtctatatcatggcc-BHQ-1 [22]. JEV primers and probe were specific for the 3′ NTR region: forward primer 5′-GGTGTAAGGACTAGAGGTTAGTGG-3′, reverse primer 5′-ATTCCCAGGTGTCAATATGCTGTT-3′ and probe FAM-cccgtggaaacaacatcatgcggc-TAMRA [23]. Reference dye was ROX (Life technologies). Real-time RT-PCR program was as follow: reverse transcription for 30 min at 50 °C; inactivation of reverse transcriptase and activation of DNA polymerase for 5 min at 95 °C; 50 cycles of denaturation for 15 s at 95 °C and annealing for 30 s at 60 °C and elongation for 30 s at 72 °C. Real-time PCR was performed with the SuperScript III Platinum One-Step qRT-PCR System (Life technologies) using 7500 Real-time PCR system (Applied Biosystems).

### Statistical analysis

Significant differences were determined with GraphPad Prism 6 software (GraphPad software Inc., La Jolla, CA) using the student *t*-Test (*P* < 0.05).

## Results

### JEV is not cytopathogenic to human microglia

**Fig. 1:**
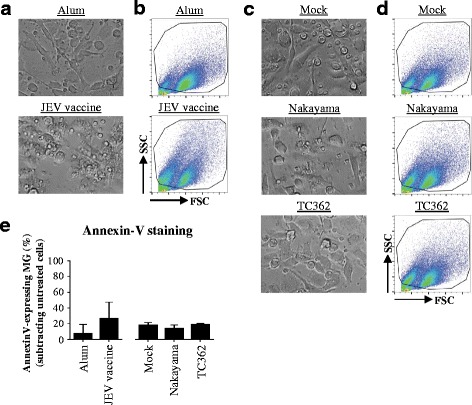
Morphology and cell death of JEV-treated human microglia. Human M-MG were treated with (**a**, **b** and **e**) Alum, JEV vaccine (used at a concentration of 1.2 pg/cell), (**c**, **d** and **e**) Mock antigen, Nakayama and TC362 isolates (used at a multiplicity of infection (MOI) of 10 TCID_50_/cell) at 37 °C for 24 h. Cell morphology and cell death were investigated. **a**, **c** Bright field micrographs (magnification of 20x) from a representative experiment of 3 independent experiments. **b**, **d** Flow cytometry analysis showing representative pseudo-colour plots of SSC versus FSC profile of human microglia observed in (**a** and **c**). Black gate delineates microglia cells excluding other cell types and cell debris. **e** Histogram bars presenting the levels of Annexin-V^+^-human microglia. Data are of 2 independent experiments with each condition performed in triplicate cultures. The bars represent the mean value; the error bars the standard deviation. Asterisks show significant differences between Alum and JEV vaccine or between Mock and the indicated live JEV isolate, calculated with the student *t*-test (* : *p* < 0.05; ** : *p* < 0.01; *** : *p* < 0.001)

A study using mouse microglia suggest that those cells are a possible viral reservoir and consequently contribute substantially to JEV pathogenesis [17]. In order to investigate the interactions between microglia in humans and JEV, changes in the morphology of the cells were explored using bright field microscopy and flow cytometry. Under the light microscope, Alum-treated human microglia presented cellular processes with a uniform cytoplasmic content whereas JEV vaccine exposure led to an amoeboid shape and the presence of large intracellular vacuoles of various sizes in human microglia (Fig. 1a). Changes in morphology were confirmed by flow cytometry (Fig. 1b). Importantly, no major changes of the morphology of human microglia treated with either the live JEV Nakayama or TC362 isolate at a multiplicity of infection (MOI) of 10 tissue culture infectious dose (TCID)_50_/cell were observed (Fig. 1c and d). Then, the viability of cells was measured in order to evaluate whether JEV induced cytotoxicity to human microglia. Flow cytometry analysis of cell surface externalization of phosphatidylserine indicated by Annexin-V staining was used to measure apoptotic cells in culture. JEV vaccine had a tendency to increase the percentage of Annexin-V^+^-microglia in comparison with the control (Fig. 1e), but results were statistically not significant. Both live Nakayama and TC362 isolates did not change the frequency of Annexin-V^+^-microglia in comparison with the mock control (Fig. 1e). Overall, live JEV does not alter the viability of human microglia in vitro.

### Chemokines secretion of Japanese encephalitis virus-treated human microglia varies between different virus isolates

**Fig. 2:**
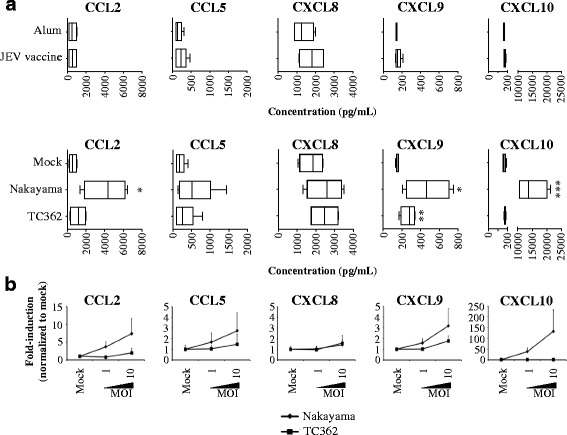
Chemokine responses of JEV-treated human microglia. Human M-MG were treated with (**a** upper panel) Alum, JEV vaccine (used at a concentration of 1.2 pg/cell), (**a** lower panel, **b**) Mock antigen, Nakayama and TC362 isolates (used at an MOI of 10 TCID_50_/cell otherwise indicated) at 37 °C for 24 h. Concentration of IL-1β and IFN-β as well as CCL2, CCL5, CXCL8, CXCL9 and CXCL10 were measured in supernatants. **a** Box plots for concentrations of the selected cytokines and chemokines. The black line represents the mean value; the error bars the standard deviation. **b** Curve line showing the effect of various doses of JEV on cytokines and chemokine ligands production. The marker represents the mean value and the error bar the standard deviation. Values are of 2 independent experiments with each condition performed in triplicate cultures. Asterisks show significant differences between Alum and JEV vaccine or between Mock and the indicated live JEV isolate, calculated with the student *t*-test (* : *p* < 0.05; ** : *p* < 0.01; *** : *p* < 0.001)

Cerebrospinal fluids of JEV-infected humans contain CCL5 and CXCL8 proteins [24]. In the brain, mRNA levels of CCL2, CCL3, CCL4 and CXCL10 are higher in JEV-infected mice than in control mice [25]. In order to determine the contribution of human microglia in inflammatory chemokine responses in JEV infection, levels of CCL2, CCL5, CXCL8, CXCL9 and CXCL10 were measured in supernatants using a chemokine beads assay and flow cytometry. Untreated human microglia demonstrated a constitutive production of chemokines that did not alter upon treatment with Alum or Mock (data not shown). In addition, treatment of cells with JEV vaccine did not affect chemokines’ production. In contrast, live Nakayama isolate at an MOI of 10 TCID_50_/cell, enhanced the production of CCL2, CXCL9 and CXCL10, but not of CCL5 and CXCL8. At a similar MOI, live TC362 isolate exclusively increased levels of CXCL9 (Fig. 2a). Moreover, chemokine responses of live JEV-treated human microglia were viral dose-dependent (Fig. 2b). However, JEV did not induce IFN-β and influence IL-1β production in human microglia cultures (data not shown). To conclude, live JEV is able to modulate the production of inflammatory chemokines in human microglia in a dose-dependent.

### Japanese encephalitis virus enhances CX_3_CR1 expression in human microglia

In various flavivirus infections such as for dengue and JEV, chemokine receptors have been shown to be critical in the disease outcome [26, 27]. In order to explore the influence of JEV on the expression of chemokine receptors on human microglia, modifications in the expression of chemokine receptors of the CC, CXC and CX_3_C sub-families were investigated using flow cytometry. After 24 h of exposure to JEV vaccine, the expressions of CCR1, CCR2, CCR3, CCR4, CCR5, CCR7, CXCR1, CXCR4 and CX_3_CR1 were modified on both human M-MG and BdMG in comparison with control cells; CXCR5 was altered on BdMG but not M-MG (Additional file 1: Figure S1). This supported the validity of the model of in-vitro generated microglia in comparison to primary cells.

**Fig. 3:**
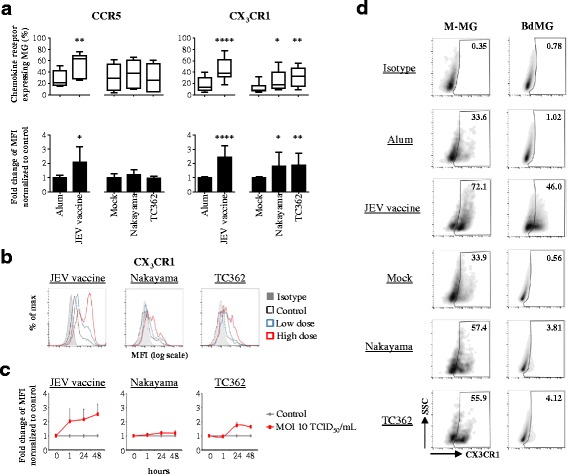
Impact of JEV on CX_3_CR1 expression in human microglia. Human microglia were treated with Alum, JEV vaccine (used at a concentration of 1.2 pg/cell otherwise indicated), Mock antigen, Nakayama and TC362 isolates (used at an MOI of 10 TCID_50_/cell otherwise indicated) at 37 °C for 24 h otherwise indicated. Expression of indicated chemokine receptor on human microglia (gated as in Fig. 1) was investigated using flow cytometry. **a** Upper panels are box plots of frequencies of the indicated chemokine receptor-expressing human microglia. The black line represents the mean value and the error bars the standard deviation. Lower panels are histogram bars of fold change in expression for the indicated chemokine receptor in human microglia. The bar represents the mean value; the error bars the standard. Values are of 3 independent experiments with each condition performed in triplicate cultures. **b** Representative histogram plots showing the effect of two different doses of JEV on CX_3_CR1 expression on human microglia. Low and high doses are respectively a concentration of 0.6 and 1.2 pg/cell in the context of JEV vaccine or an MOI of 1 and 10 TCID_50_/cell in the context of live JEV. **c** Curve lines of fold change in expression for CX_3_CR1 in human microglia upon treatment with JEV for various time periods. The marker represents the mean value and the error bar the standard deviation. Values are of a representative experiment with each condition performed in triplicate cultures. **d** Left panel displays representative SSC versus CX_3_CR1 density plots of human M-MG treated with a concentration 1.2 pg/cell in the context of JEV vaccine or an MOI of 10 TCID_50_/cell in the context of live JEV. Right panel displays representative SSC versus CX_3_CR1 density plots of human BdMG treated with a concentration 1.2 pg/cell in the context of JEV vaccine or an MOI of 0.1 TCID_50_/cell in the context of live JEV. The black gate frames the CX_3_CR1^+^ human microglia population and frequencies are indicated. Asterisks show significant differences between Alum and JEV vaccine or between Mock and the indicated live JEV isolate, calculated with the student *t*-test (* : *p* < 0.05; ** : *p* < 0.01; *** : *p* < 0.001)

Expressions of CCR5 and CX3CR1 on untreated human microglia were not significantly modified by treatment with Alum or Mock (data not shown). Significantly, both the frequencies and levels of expression of CCR5 and CX3CR1 on M-MG were higher upon treatment with JEV vaccine. At an MOI of 10 TCID_50_/cell, both Nakayama and TC362 isolates modified CX_3_CR1 but not CCR5 on M-MG, in terms of both frequencies and levels of expression of the chemokine receptor (Fig. 3a). In addition, higher doses of JEV led to higher expression of CX_3_CR1 on human microglia (Fig. 3b). While CX_3_CR1 expression on JEV vaccine-treated cells was up regulated as fast as 1 h and stabilized up to 48 h, live JEV induced transient up-regulation of CX_3_CR1 expression, with a peak at 24 h (Fig. 3c). Importantly, primary human microglia also demonstrated increased frequencies of CX_3_CR1^+^-cells upon treatment with inactivated and live JEV. Interestingly, an MOI of 0.1 TCID_50_/cell of live JEV was necessary to modify CX_3_CR1 expression on BdMG (Fig. 3d). Higher MOIs did not affect CX_3_CR1 expression on BdMG (data not shown). Overall, JEV significantly enhanced CX_3_CR1 expression on human microglia in a dose- and a time-dependent manner.

### Both JEV vaccine and live JEV enhance MHCII expression on CX_3_CR1^+^ human microglia

**Fig. 4:**
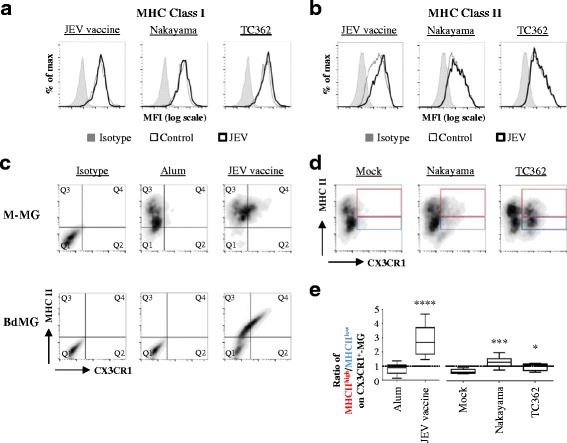
Impact of JEV on MHC expression in human microglia. Human microglia were treated with Alum, JEV vaccine (used at a concentration of 1.2 pg/cell), Mock antigen, Nakayama and TC362 isolates (used at an MOI of 10 TCID_50_/cell) at 37 °C for 24 h. Expression of indicated MHC molecules on human microglia (gated as in Fig. 1) was investigated using flow cytometry. **a**, **b** Representative histogram plots showing the effect of JEV on MHC (**a**) class I and (**b**) class II. **c** Representative MHC class II versus CX_3_CR1 density plots of human M-MG (upper panel) and BdMG (lower panel) treated with JEV vaccine. **d** Representative MHC class II versus CX_3_CR1 density plots of human M-MG treated with live JEV. **e** Box plots showing the ratio of MHCII^high^ versus MHCII^low^ on CX_3_CR1^+^-human M-MG subset. The black line represents the mean value and the error bars the standard deviation. Asterisks show significant differences between Alum and JEV vaccine or between Mock and the indicated live JEV isolate, calculated with the student *t*-test (* : *p* < 0.05; ** : *p* < 0.01; *** : *p* < 0.001)

In the CNS, microglial cells link the innate and the adaptive immunity by presenting MHC molecules loaded with exogenous antigen to T-cells. Microglia cells have a constitutive, but low expressions of MHC class I and II that increase in response to insults [15]. Consequently, the effect of JEV on the expression of MHC class I and II on human microglia was assessed using flow cytometry. No differences in MHCI expression were perceived on M-MG upon treatment with JEV vaccine and live JEV (Fig. 4a). While MHCII expression on M-MG was enhanced upon treatment with JEV vaccine, no modifications were observed if treated with live JEV isolates (Fig. 4b).

Since live JEV was inefficient to influence the expression of MHCII in the total microglia cell population, further study focused on the impact of JEV on MHCII expression in various subsets of human microglia co-stained for MHCII and CX_3_CR1 before flow cytometry analysis. Control M-MG had low levels of expression for both MHCII and CX_3_CR1 whereas control BdMG were negative for both CX_3_CR1 and MHCII. Upon exposure to JEV vaccine, both M-MG and BdMG were of two major microglia subsets: one was CX_3_CR1^-^/MHCII^+^ (Fig. 4c: quadrant Q3) and another was CX_3_CR1^+^/MHCII^+^ (Fig. 4c: quadrant Q4). Because M-MG showed stronger responsiveness than BdMG to JEV exposure, further analysis were done using M-MG. In order to identify the most susceptible human microglia subset to JEV exposure, the frequencies of the CX_3_CR1^+^/MHCII^high^ (Fig. 4d: red gate) and the CX_3_CR1^+^/MHCII^low^ (Fig. 4d: blue gate) subsets were compared. In control cells, the ratio of CX_3_CR1^+^/MHCII^high^ over CX_3_CR1^+^/MHCII^low^ was low with values of 0.89 (±.0.3) and 0.69 (±.0.17) upon exposure to alum and mock antigen respectively (Fig. 4e). This detailed analysis revealed that upon exposure to JEV vaccine, in particular, but also to both Nakayama and TC362 live isolates at an MOI of 10 TCID_50_/cell, the ratio of CX_3_CR1^+^/MHCII^high^ over CX_3_CR1^+^/MHCII^low^ significantly increased with values of 2.57 (±.0.88),1.25 (±.0.36) and 0.86 (±.0.26), respectively (Fig. 4e). To conclude, JEV led to co-enhancement of CX_3_CR1 and MHCII on a subpopulation of human microglia.

### Human microglia support JEV replication and virus transmission to susceptible cells

**Fig. 5:**
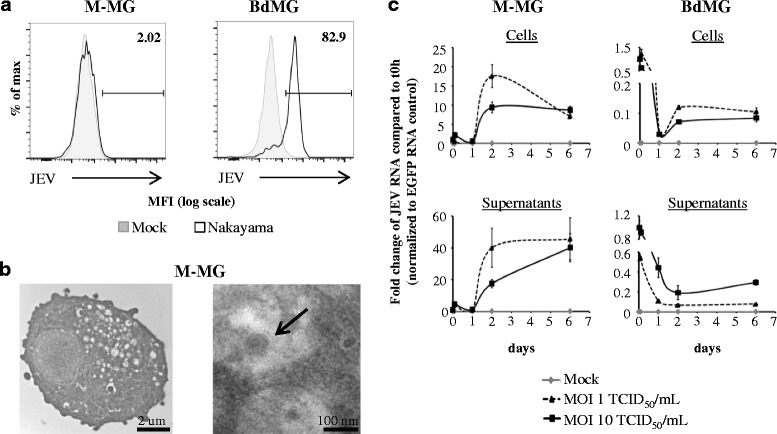
JEV detection and replication in human microglia. Human microglia were treated with Mock antigen and Nakayama isolate (used at an MOI of 10 TCID_50_/cell otherwise indicated) at 37 °C for various time periods. Virus detection and viral replication were evaluated. **a** Representative histogram plots of flow cytometry analysis showing intracellular JEV particles in human microglia (as gated in Fig. 1) after 48 h of exposure. Frequencies of JEV^+^-human microglia are indicated. **b** Transmission electron micrographs of Nakayama-treated human M-MG (right panel, magnification of 9700x) containing JEV particles (left panel, magnification of 66000x) after 24 h of exposure. Bars indicate the scale. Images are from a representative experiment of 2 independent experiments. **c** Curve lines of fold change in viral RNA compared to the time point 0 h using real-time RT-PCR. The marker represents the mean value and the error bar the standard deviation. Values are of a representative experiment with each condition performed in triplicate reaction

Mouse microglia are productively infected by JEV [17]. Therefore, the infection of human microglia by Nakayama isolate was evaluated using flow cytometry and transmission electron microscopy. Over 80% of BdMG stained for intracellular virus, whereas less than 2.5% of M-MG were positive for intracellular virus detection (Fig. 5a). Importantly, intracellular vacuoles and icosahedral JEV particles of ~50 nm diameter were visible in M-MG using transmission electron microscopy (Fig. 5b).

In order to evaluate the support of human microglia in JEV propagation, viral RNA and infectious particles were analysed in such cultures. First, JEV replication in human microglia was assessed by quantification of viral RNA in cells and supernatants using real-time RT-PCR. In order to detect *de novo* synthesis of JEV RNA, unbound virus was washed away after a 2 h step of virus attachment on cells. Then, viral RNA was measured over a time period of 6 days post-infection (p.i). In both M-MG and BdMG cell extracts, the eclipse phase of virus replication was characterized by a drop of viral RNA during the first 24 h of infection. This was followed by the exponential phase of virus replication to reach a peak at 2 days p.i. Viral RNA was detected up to 6 days p.i. (Fig. 5c, upper panels). In parallel, viral RNA was measured in supernatants to determine whether JEV RNA was released. In supernatants, an eclipse phase was observed during the first 24 h and 48 h in M-MG and BdMG, respectively. Then, viral RNA in supernatants increased and was detected at 6 days p.i, in both M-MG and BdMG cultures (Fig. 5c, lower panels). Interestingly, M-MG allowed higher fold change of JEV RNA compared to time point 0 h than BdMG (Fig. 5c).

**Fig. 6:**
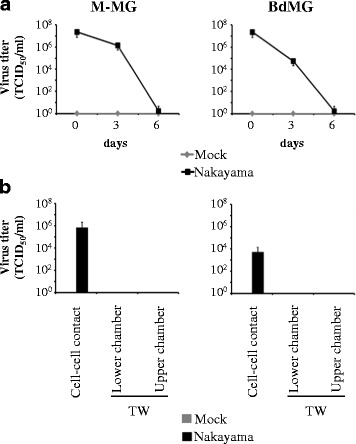
Infectiousness of JEV-infected human microglia. Human microglia were treated with Mock antigen and Nakayama isolate (used at an MOI of 10 TCID_50_/cell) at 37 °C for various time periods. **a** Infectious JEV particles were determined by endpoint titration in supernatants of cultures. Curve line representing infectious virus titre over time in supernatants of human microglia cultures. The marker represents the mean value and the error bar the standard deviation. **b** Histogram bars representing infectious virus titre in supernatants of 6 days Nakayama-infected human microglia cultured with BHK-21 for 2 days, without (cell-cell contact) or with transwell insert (TW). The bar represents the mean value; the error bars the standard. Values are of 3 independent experiments

Since viral RNA was detected in both supernatants and cells, the infectiousness of JEV-derived human microglia was tested. Surprisingly, titers of infectious JEV decreased in supernatants of both M-MG and BdMG cultures over time and no infectious JEV was found after 6 days of exposure (Fig. 6a). Since supernatants of JEV-derived human microglia were not infectious, the infectiousness of cell-associated JEV was investigated. To this end, 6 days Nakayama-infected human microglia were cultured in presence of BHK-21 cells for 2 days and infectious JEV derived from BHK-21 was measured in supernatant. On one hand, if human microglia and BHK-21 cells were cultured in cell-cell contact condition, *de novo* infectious virus reached titres of ~10^5^ TCID_50_/mL and ~10^3^ TCID_50_/mL for M-MG/BHK-21 and BdMG/BHK-21 cultures, respectively. On another hand, if human microglia and BHK-21 cells were separated using transwell insert (TW), no infectious JEV was detected in supernatants of both the lower chamber containing human microglia and the upper chamber containing BHK-21 cells (Fig. 6b). In conclusion, although human microglia do not release infectious virus, they can transmit JEV to neighbouring cells in a cell-cell contact manner.

## Discussion

Interactions between JEV and microglial cells have been demonstrated in various species including rats, mice and macaques. Up to date, interactions between JEV and human microglia were shown using cell lines. Here, human primary microglia isolated from brains of cadavers and an in-vitro model of human blood monocytes-derived microglia were employed. Although microglia originate from the yolk sack during embryogenesis, monocytes can contribute to the microglial cell population after birth, justifying the use of this culture model [13, 14]. Furthermore, in West Nile virus (WNV)-infected mice, CNS-infiltrating inflammatory monocytes have been shown to be microglia precursors [28]. Our data demonstrate that primary and in-vitro generated human microglia show differences in sensitivity to JEV exposure, in terms of E protein expression, but both human microglia models supported viral propagation and shared the overall chemokine receptor pattern. Therefore, human blood monocyte-derived microglia represent a valid model to study microglia-related disease pathogenesis, such as for JE.

JE is characterized by uncontrolled inflammatory responses in the periphery and in the CNS. JEV-infected patients present CSF-derived CCL5 and CXCL8 [24] and chemokines such as CC2, CCL3, CCL4 and CXCL10 are expressed in brains of JEV-infected mice [25]. In the brain, microglia is one source of cytokines and chemokines including interleukin (IL)-6, IL-1β, CCL2 and CCL5 in various species including mice and primate models [29–32]. Here, JEV-exposed microglia had enhanced production of CCL2, CXCL9 and CXCL10, indicating the possible contribution of human microglia in the presence of pro-inflammatory chemokine in the brain compartment of JE patients. Nevertheless, chemokines may have either detrimental or beneficial impact for the outcome of the disease. For example, the interaction of CCL2 with CCR2 orchestrates the recruitment of inflammatory monocytes into the brain of WNV-infected mice in a pathogenic manner [28]. In contrast, the signalling CXCL10/CXCR3 is implicated in the infiltration of virus-specific CD8^+^ T lymphocytes into the brain leading to a prolong survival of WNV-infected mice [33]. In JEV-infected mice, CD8^+^ T lymphocytes accumulate in the brain [27] and the activity of cytotoxic lymphocytes partly mediates protection against JEV [34]. Indeed, impaired activity and trafficking of CD8^+^ T-cell into the CNS contribute to increased mortality of CCR5-deficient mice exposed to JEV [27]. With our data in mind, also in human, microglia-derived CCL2, CXCL9 and CXCL10 may contribute to leukocyte trafficking into the CNS, required for JE recovery.

Interestingly, exposure of human microglia to different JEV isolates led to differences in the level and the signature of chemokine responses. These differences may be explained by differential binding and entry efficiency of the viruses to the host cell. JEV interacts with its still unidentified host cell receptor via the viral envelope E protein [35]. Modifications of the E protein can alter JEV binding and penetration into the target cell [36]. Another explanation could be related to the activation of specific pattern recognition receptors (PRR) upon JEV infection. Interestingly, the activation of certain PRRs leads to contrasting regulation of JE. Indeed, Toll-like receptor (TLR)3^-/-^ but not TLR4^-/-^ mice are highly susceptible to JE characterized by severe CNS inflammation [37]. In microglia, both the endosomal TLR3 and the cytosolic retinoic acid-inducible gene 1 (RIG-I) sense the presence of JEV. Interestingly, JEV-induced CCL2 production has been shown to be abrogated in microglia of RIG-I-, but not in TLR3-knockdown mice [32]. Although multiple PRR are able to sense the presence of JEV, the cellular localization of virus particles and the activation of specific PRRs seem to be crucial to generate an efficient and non-pathogenic inflammatory response.

A histological study of the brain in JEV-infected macaques showed that activated microglia cells express MHC class II [31]. Our results revealed a tendency of expression of MHC class II on activated CX_3_CR1^+^-human microglia upon exposure to JEV. MHC class II is also increased on JEV-exposed human MoDC, but not MDM [11, 12]. MHC class II is involved in the reactivation of virus-specific CD4^+^ T-lymphocytes. Helper T-cells accumulate in the CNS of JEV-infected mice [27] and are a critical source of IFN-γ which promotes JEV clearance [34]. In this context, it was interesting to note that human microglia exposed to live JEV showed weak modification of MHC class II on CX_3_CR1^+^ microglia. This would limit their ability to present antigen to local virus-specific CD4^+^ T-cells.

CX_3_CR1 was consistently up-regulated on JEV-exposed human microglia. Since CX_3_CR1 is mainly expressed by microglia and its unique ligand CX_3_CL1 is primarily expressed by neurons [38, 39], the CX_3_CL1/CX_3_CR1 signalling is central for microglia-neuron interactions regulating neuroinflammation, neuroprotection as well as chemotaxis. CX_3_CL1 inhibits the production of pro-inflammatory cytokines by microglia [40] which could control neurotoxicity of JEV-infected microglia-derived inflammatory factors in mice [29]. CX_3_CL1 also mediates chemo-attraction of CX_3_CR1-expressing microglia [38]. JEV-exposed human microglia expressed CX_3_CR1 and were able to transmit JEV to susceptible cells. Moreover, the virus transmission happened in a cell-cell contact manner. It is thus possible to imagine that a gradient of CX_3_CL1 could attract JEV-infected CX_3_CR1^+^-microglia allowing further JEV transmission to neuronal cells, the major target cells of JEV.

A possible role of microglia as reservoir for JEV has been proposed using a mouse microglia cell line [17]. In our study, we show that human microglia supported JEV replication without cytopathogenic effects and cell-associated viral particles remained infectious. JEV-infected microglia may be involved in infection of neurons, contributing to JEV pathogenesis.

## Conclusion

Taken together, the understanding of the role of microglia in JEV pathogenesis may be helpful to identify therapeutic targets for JE patients. Dissecting pathogenic from protective microglia responses might help to understand the pathogenesis of severe virus-induced encephalitis and identify possible strategies of a therapeutic manipulation of microglia responses. In particular, the signalling CX_3_CL1/CX_3_CR1 represents a potential target for inflammatory diseases [41], such as for JE.

## Additional file

Additional file 1: Figure S1. JEV vaccine-induced chemokine receptor pattern in human microglia. Human M-MG and BdMG were treated with Alum and JEV vaccine (used at a concentration of 1.2 pg/cell) at 37 °C for 24 h. Cells were stained for the indicated chemokine receptor and analysed by flow cytometry. Representative histogram plots of (A) CC, (B) CXC and (C) CX3C chemokine receptor expressing-human M-MG (upper panel) and BdMG (lower panels) are shown. Cells were gated as in Fig. 1. (PDF 208 kb)

## Abbreviations

Alum: Aluminium hydroxide in suspension
BdMG: Human primary microglia
CNS: Central nervous system
JE: Japanese encephalitis
JEV: Japanese encephalitis virus
M-MG: Human monocyte-derived microglia
MOI: Multiplicity of infection

## References

[1] Misra UK, Kalita J (2010). Overview: Japanese encephalitis. Prog Neurobiol.

[2] Solomon T (2004). Flavivirus encephalitis. N Engl J Med.

[3] Huber K, Jansen S, Leggewie M, Badusche M, Schmidt-Chanasit J, Becker N (2014). Aedes japonicus japonicus (Diptera: Culicidae) from Germany have vector competence for Japan encephalitis virus but are refractory to infection with West Nile virus. Parasitol Res.

[4] Ricklin ME, Garcia-Nicolas O, Brechbuhl D, Python S, Zumkehr B, Nougairede A (2016). Vector-free transmission and persistence of Japanese encephalitis virus in pigs. Nat Commun.

[5] Campbell GL, Hills SL, Fischer M, Jacobson JA, Hoke CH, Hombach JM (2011). Estimated global incidence of Japanese encephalitis: a systematic review. Bull World Health Organ.

[6] van den Hurk AF, Ritchie SA, Mackenzie JS (2009). Ecology and geographical expansion of Japanese encephalitis virus. Annu Rev Entomol.

[7] Swarup V, Ghosh J, Das S, Basu A (2008). Tumor necrosis factor receptor-associated death domain mediated neuronal death contributes to the glial activation and subsequent neuroinflammation in Japanese encephalitis. Neurochem Int.

[8] Kimura-Kuroda J, Ichikawa M, Ogata A, Nagashima K, Yasui K (1993). Specific tropism of Japanese encephalitis virus for developing neurons in primary rat brain culture. Arch Virol.

[9] Desai A, Shankar SK, Ravi V, Chandramuki A, Gourie-Devi M (1995). Japanese encephalitis virus antigen in the human brain and its topographic distribution. Acta Neuropathol.

[10] Yang KD, Yeh WT, Chen RF, Chuon HL, Tsai HP, Yao CW (2004). A model to study neurotropism and persistency of Japanese encephalitis virus infection in human neuroblastoma cells and leukocytes. J Gen Virol.

[11] Gupta N, Hegde P, Lecerf M, Nain M, Kaur M, Kalia M (2014). Japanese encephalitis virus expands regulatory T cells by increasing the expression of PD-L1 on dendritic cells. Eur J Immunol.

[12] Sooryanarain H, Sapkal GN, Gore MM (2012). Pathogenic and vaccine strains of Japanese encephalitis virus elicit different levels of human macrophage effector functions. Arch Virol.

[13] Mildner A, Schmidt H, Nitsche M, Merkler D, Hanisch UK, Mack M (2007). Microglia in the adult brain arise from Ly-6ChiCCR2+ monocytes only under defined host conditions. Nat Neurosci.

[14] Ginhoux F, Lim S, Hoeffel G, Low D, Huber T (2013). Origin and differentiation of microglia. Front Cell Neurosci.

[15] Yang I, Han SJ, Kaur G, Crane C, Parsa AT (2010). The role of microglia in central nervous system immunity and glioma immunology. J Clin Neurosci.

[16] Ghoshal A, Das S, Ghosh S, Mishra MK, Sharma V, Koli P (2007). Proinflammatory mediators released by activated microglia induces neuronal death in Japanese encephalitis. Glia.

[17] Thongtan T, Cheepsunthorn P, Chaiworakul V, Rattanarungsan C, Wikan N, Smith DR (2010). Highly permissive infection of microglial cells by Japanese encephalitis virus: a possible role as a viral reservoir. Microbes Infect/Institut Pasteur.

[18] Melief J, Koning N, Schuurman KG, Van De Garde MD, Smolders J, Hoek RM (2012). Phenotyping primary human microglia: tight regulation of LPS responsiveness. Glia.

[19] Etemad S, Zamin RM, Ruitenberg MJ, Filgueira L (2012). A novel in vitro human microglia model: characterization of human monocyte-derived microglia. J Neurosci Methods.

[20] Duggan ST, Plosker GL (2009). Japanese encephalitis vaccine (inactivated, adsorbed) [IXIARO]. Drugs.

[21] Lannes N, Summerfield A (2013). Regulation of porcine plasmacytoid dendritic cells by cytokines. PLoS One.

[22] Hoffmann B, Depner K, Schirrmeier H, Beer M (2006). A universal heterologous internal control system for duplex real-time RT-PCR assays used in a detection system for pestiviruses. J Virol Methods.

[23] Yang DK, Kweon CH, Kim BH, Lim SI, Kim SH, Kwon JH (2004). TaqMan reverse transcription polymerase chain reaction for the detection of Japanese encephalitis virus. J Vet Sci.

[24] Winter PM, Dung NM, Loan HT, Kneen R, Wills B, le Thu T (2004). Proinflammatory cytokines and chemokines in humans with Japanese encephalitis. J Infect Dis.

[25] Yang Y, Ye J, Yang X, Jiang R, Chen H, Cao S (2011). Japanese encephalitis virus infection induces changes of mRNA profile of mouse spleen and brain. Virol J.

[26] Guabiraba R, Marques RE, Besnard AG, Fagundes CT, Souza DG, Ryffel B (2010). Role of the chemokine receptors CCR1, CCR2 and CCR4 in the pathogenesis of experimental dengue infection in mice. PLoS One.

[27] Larena M, Regner M, Lobigs M (2012). The chemokine receptor CCR5, a therapeutic target for HIV/AIDS antagonists, is critical for recovery in a mouse model of Japanese encephalitis. PLoS One.

[28] Getts DR, Terry RL, Getts MT, Muller M, Rana S, Shrestha B (2008). Ly6c + “inflammatory monocytes” are microglial precursors recruited in a pathogenic manner in West Nile virus encephalitis. J Exp Med.

[29] Das S, Mishra MK, Ghosh J, Basu A (2008). Japanese Encephalitis Virus infection induces IL-18 and IL-1beta in microglia and astrocytes: correlation with in vitro cytokine responsiveness of glial cells and subsequent neuronal death. J Neuroimmunol.

[30] Chen CJ, Ou YC, Lin SY, Raung SL, Liao SL, Lai CY (2010). Glial activation involvement in neuronal death by Japanese encephalitis virus infection. J Gen Virol.

[31] Myint KS, Kipar A, Jarman RG, Gibbons RV, Perng GC, Flanagan B (2014). Neuropathogenesis of Japanese encephalitis in a primate model. PLoS Negl Trop Dis.

[32] Jiang R, Ye J, Zhu B, Song Y, Chen H, Cao S (2014). Roles of TLR3 and RIG-I in mediating the inflammatory response in mouse microglia following Japanese encephalitis virus infection. J Immunol Res.

[33] Klein RS, Lin E, Zhang B, Luster AD, Tollett J, Samuel MA (2005). Neuronal CXCL10 directs CD8+ T-cell recruitment and control of West Nile virus encephalitis. J Virol.

[34] Larena M, Regner M, Lobigs M (2013). Cytolytic effector pathways and IFN-gamma help protect against Japanese encephalitis. Eur J Immunol.

[35] Zu X, Liu Y, Wang S, Jin R, Zhou Z, Liu H (2014). Peptide inhibitor of Japanese encephalitis virus infection targeting envelope protein domain III. Antivir Res.

[36] Liu H, Chiou SS, Chen WJ (2004). Differential binding efficiency between the envelope protein of Japanese encephalitis virus variants and heparan sulfate on the cell surface. J Med Virol.

[37] Han YW, Choi JY, Uyangaa E, Kim SB, Kim JH, Kim BS (2014). Distinct dictation of Japanese encephalitis virus-induced neuroinflammation and lethality via triggering TLR3 and TLR4 signal pathways. PLoS Pathog.

[38] Harrison JK, Jiang Y, Chen S, Xia Y, Maciejewski D, McNamara RK (1998). Role for neuronally derived fractalkine in mediating interactions between neurons and CX3CR1-expressing microglia. Proc Natl Acad Sci U S A.

[39] Hatori K, Nagai A, Heisel R, Ryu JK, Kim SU (2002). Fractalkine and fractalkine receptors in human neurons and glial cells. J Neurosci Res.

[40] Zujovic V, Benavides J, Vige X, Carter C, Taupin V (2000). Fractalkine modulates TNF-alpha secretion and neurotoxicity induced by microglial activation. Glia.

[41] Jones BA, Beamer M, Ahmed S (2010). Fractalkine/CX3CL1: a potential new target for inflammatory diseases. Mol Interv.

