# Supplementary figures and images for: Interactions of human microglia cells with Japanese encephalitis virus
Source: Virol J. 2017 Jan 14;14:8. doi: 10.1186/s12985-016-0675-3 (PMC5237516; doi:10.1186/s12985-016-0675-3)

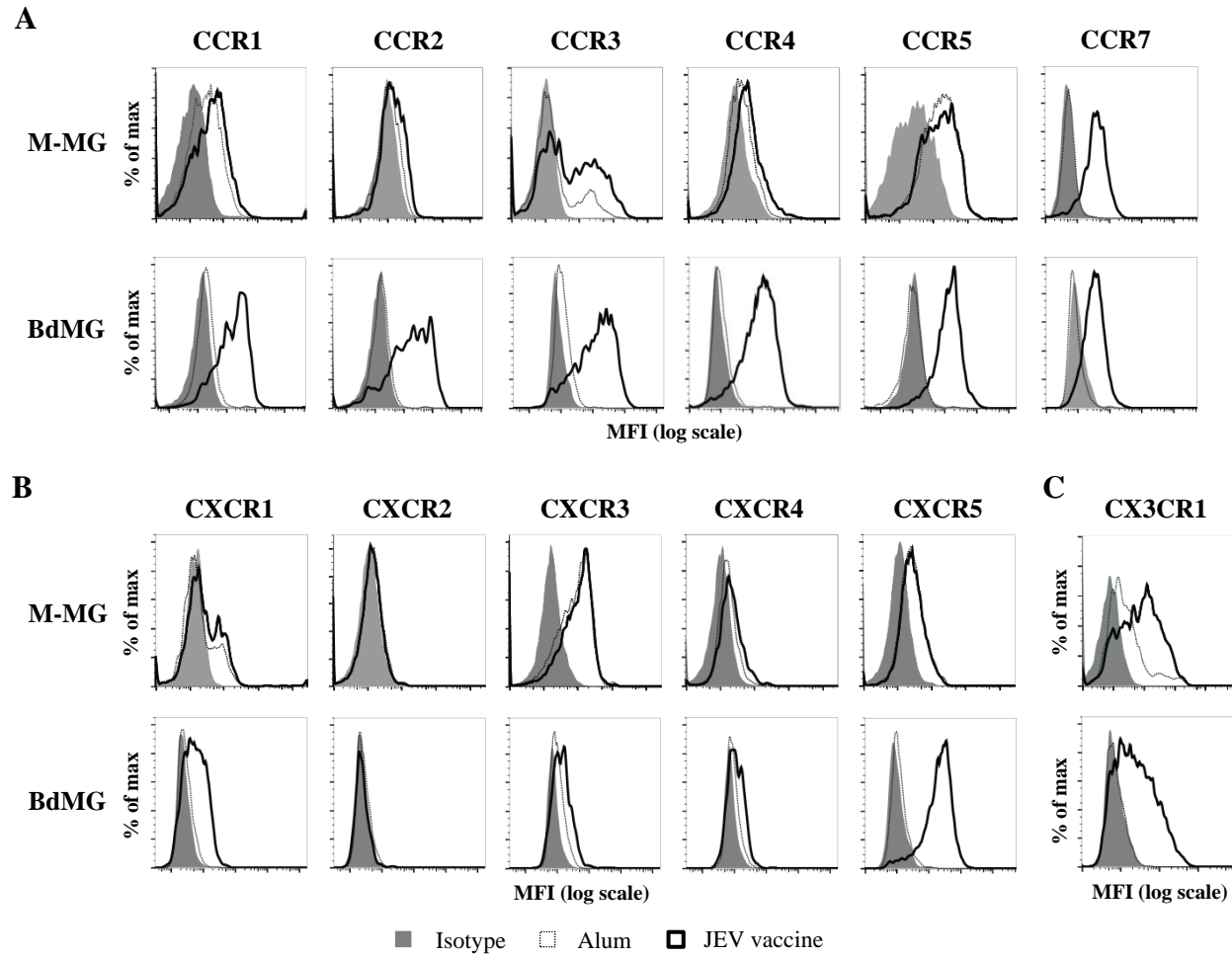

Supplement: Additional file 1: Figure S1. — JEV vaccine-induced chemokine receptor pattern in human microglia. Human M-MG and BdMG were treated with Alum and JEV vaccine (used at a concentration of 1.2 pg/cell) at 37 °C for 24 h. Cells were stained for the indicated chemokine receptor and analysed by flow cytometry. Representative histogram plots of (A) CC, (B) CXC and (C) CX3C chemokine receptor expressing-human M-MG (upper panel) and BdMG (lower panels) are shown. Cells were gated as in Fig. 1. (PDF 208 kb) [file 12985_2016_675_MOESM1_ESM.pdf]
